# Large‐Scale Protein Assay Identifies Novel Protein Biomarkers Associated With Arterial Stiffness and Vascular Calcification Measures

**DOI:** 10.1155/ijhy/9724102

**Published:** 2026-07-14

**Authors:** Rain Katz, Ryan Cvejkus, Jiebiao Wang, Bharat Thyagarajan, Emma Barinas-Mitchell, John Jeffrey Carr, James G. Terry, Sangeeta Nair, Winnie Wan-Yee Tang, Rachel G. Miller, Iva Miljkovic, Joseph M. Zmuda, Allison L. Kuipers

**Affiliations:** ^1^ Department of Epidemiology, University of Pittsburgh School of Public Health, 130 De Soto St, Pittsburgh, 15261, Pennsylvania, USA, ufl.edu; ^2^ Department of Biostatistics, University of Pittsburgh School of Public Heatlth, 130 De Soto St, Pittsburgh, 15261, Pennsylvania, USA; ^3^ Department of Laboratory Medicine and Pathology, University of Minnesota Medical School, 420 Delaware Street SE, Minneapolis, 55455, Minneapolis, USA, umn.edu; ^4^ Division of Cardiovascular Medicine, Vanderbilt University Medical Center, 1161 21st Ave S, Nashville, Tennessee, 37232, USA, vanderbilt.edu; ^5^ Department of Environmental and Occupational Health, University of Pittsburgh School of Public Health, 130 De Soto St, Pittsburgh, 15261, Pennsylvania, USA; ^6^ Departments of Medicine, and Epidemiology and Biostatistics, Michigan State University, 15 Michigan St NE, Grand Rapids, Michigan, 49503, USA, msu.edu; ^7^ Fredrik Meijer Heart & Vascular Institute, Corewell Health, 100 Michigan St NE, Grand Rapids, Michigan, 49503, USA

## Abstract

Large‐scale protein assays have the potential to identify novel biomarkers and improve prediction of cardiovascular disease (CVD) risk. The burden of atherosclerotic CVD and related risk factors is disproportionately high in Afro‐Caribbean populations in whom novel risk prediction strategies may be warranted. Thus, we analyzed the association of early atherosclerosis markers with peripheral blood proteomic biomarkers quantified using the Olink Target 96 Cardiovascular III panel in 342 community‐dwelling men from the Tobago Health Study. Atherosclerosis markers included pulse wave velocity (PWV), abdominal aortic calcification (AAC), and coronary artery calcification (CAC). Blood pressure, height, and weight were measured by trained clinic staff. Hypertension, dyslipidemia, and diabetes status were classified via clinical measures and the prescription of relevant medication. Statistical methods included differential expression analysis fully adjusted for assay batch, age, BMI, smoking, diabetes, dyslipidemia, and blood pressures. Men ranged from 53 to 89 years of age (mean ± SD = 63.3 ± 8.1 years). Average BMI was 27.7 ± 4.3 kg/m^2^, 79.2% had hypertension, average LDL cholesterol was 129.4 ± 37.7 mg/dL, 24.6% had diabetes, and 5.9% were current smokers. In fully adjusted models, 18 proteins were associated with PWV and 1 was associated with CAC. There were no significant associations with AAC. Thirteen of these significant associations (12 PWV and 1 CAC) were novel, having never been identified as associated with these atherosclerosis measures in any previous study. While these proteins are involved in physiological processes known to contribute to CVD, such as inflammation, the strongest effects were seen for previously unreported protein associations. Our work is the first large‐scale protein assay performed in an Afro‐Caribbean population and adds to existing evidence that indicates that protein biomarker signatures of CVD may differ by racial/ethnic group. Further research is needed to understand the role of protein biomarkers in the prediction and progression of CVD among this high‐risk group.

## 1. Introduction

Cardiovascular disease (CVD) is the leading cause of death both globally and in the United States. Several cardiovascular traits have been established as important early markers of atherosclerotic CVD, including arterial stiffness and vascular calcification. Pulse wave velocity (PWV) is a measure of the speed at which the pulse caused by each contraction of the heart propagates throughout the circulatory system. Carotid–femoral PWV (cfPWV) is the “gold standard” measure of arterial stiffness—the loss of elasticity in blood vessels that is related to the progression of atherosclerosis—and higher PWV has been rigorously shown to predict future CVD events and mortality [1]. The formation of calcified plaques in arteries is also a marker of atherosclerotic CVD. In particular, coronary artery calcification (CAC) and abdominal aortic calcification (AAC), which can be measured by computed tomography (CT) imaging, have been shown to predict short‐ and long‐term risk of cardiovascular events in healthy individuals [2].

Previous research has shown many single proteins to predict future CVD events, including N‐terminal pro‐B‐type natriuretic peptide (NT‐proBNP) [3], gamma glutamyltransferase (GGT) [4], homocysteine [5], troponin [6], and C‐reactive protein (CRP) [7], among many others [8]. The field of proteomics, which involves profiling many proteins simultaneously, has emerged as a method to improve prediction of CVD by gaining a deeper understanding of the biological pathways that cause CVD to develop [9]. Studying many proteins simultaneously has been effective in improving the accuracy of models that predict future CVD [10] and identifying novel biological pathways that develop CVD [11].

Proteomic analysis in the Jackson Heart Study (JHS), a US‐based study of CVD in Black Americans, was able to identify novel protein markers of CVD that had not been identified in other populations [12]. Research in the JHS has also been able to identify proteins that are capable of predicting incident coronary heart disease and are involved in biological pathways specific to Black individuals that have not been previously studied [13]. These findings demonstrate that protein/CVD relationships vary between racial groups, highlighting the need for targeted studies in unique populations. A separate study assayed a set of 47 protein markers thought to potentially predict CVD in a mixed cohort of non‐Hispanic White and African‐American individuals and found that the levels of more than half of the proteins being studied differed by race [14]. For example, the average concentration of CRP, an inflammatory marker known to be associated with CVD, was almost 40% higher in Black men compared to White men. Similar effects were observed for NT‐proBNP, tumor necrosis factors 1 and 2 (TNFR1 and TNFR2), and matrix metalloproteinase‐9 (MMP9), among many other protein markers. Together, these studies provide evidence for racial differences in protein markers associated with CVD.

Globally, populations of African ancestry, including Afro‐Caribbeans, tend to experience disproportionate rates of CVD [15]. The island nation of Trinidad and Tobago has one of the highest rates of age‐adjusted CVD morality across North and South America [16], and the Afro‐Caribbean–dominated population of Tobago in particular is known to have an extremely high prevalence of CVD risk factors such as obesity and hypertension [17]. However, the relationship between large‐scale protein assays and markers of CVD has never been studied in any Afro‐Caribbean population, and it is unknown whether differences in concentrations of protein markers may partially explain their uniquely high risk of CVD, or whether there are unique protein biomarkers that are predictive of future CVD in these populations.

Thus, we examined the association of peripheral blood proteomic biomarkers with early atherosclerosis markers in the well‐characterized Tobago Health Study (THS) [18, 19]. We hypothesize that the protein signatures associated with markers of atherosclerosis—specifically PWV, CAC, and AAC—in this population will differ from those previously identified in studies of European‐ancestry populations.

## 2. Methods

### 2.1. THS

The THS, a population‐based longitudinal cohort study of Tobagonian adults, was established to study the unique burden of chronic disease in this predominantly Afro‐Caribbean population. Screening and recruitment for the initial THS were performed between 1997 and 2003 and originally focused on prostate cancer screening and enrolled participants without regard to health status due to its screening nature. Specifically, the initial THS included men, aged 40–79 years, who were ambulatory, noninstitutionalized, and free from terminal disease. A total of 3170 men were enrolled, representing 60% of the men in the eligible age range on the island [18];thus, the THS is reflective of the general adult male population on Tobago. A follow‐up visit conducted from 2014 to 2017 included the standard THS data collection items (biological samples [blood and urine], thorough health histories [including previous diagnoses of CVD and other chronic health conditions], and clinical examinations [blood pressures, height, and weight]), and introduced measures of early atherosclerosis progression, including PWV and CT measures of AAC and CAC, in approximately 800 men. A random subset of men who had data for all atherosclerosis measures (*N* = 342) were selected for measures of fasting serum protein concentration. Covariate and outcome characteristics of the selected sample do not differ significantly from the overall 2014–2017 THS (Supporting Table 1). The Institutional Review Boards of the University of Pittsburgh and the Tobago Ministry of Health and Social Services approved all protocols associated with this study. All participants provided written informed consent before data collection.

### 2.2. Measurement of Atherosclerosis Markers

Brachial‐ankle PWV (baPWV) was measured using a noninvasive, waveform analyzer (Colin‐VP1000, Omron, Japan/WaveNexus, TX), as previously described [17]. Following 10 min of rest in a supine position, occlusion/monitoring cuffs were placed at standardized locations on lightly clothed arms and bare ankles. The cuffs were connected to a plethysmographic sensor and an oscillometric pressure sensor, which recorded volume pressure waveforms and blood pressure, respectively. The volume pressure waveforms were collected at the arm (brachial artery) and ankle (tibial artery) over a total sampling time of 10 s with automatic gain analysis and quality adjustment. baPWV was calculated as the (distance between arterial sites in cm)/(time between the foot of the waveforms in s). The distance was measured using a height‐based formula [20] that accounts for the opposite direction of blood flow by subtracting the distance from the brachial artery to the heart. Mean baPWV was used in this analysis as the average of four measures, two each on the left and right sides of the body.

Vascular calcification was assessed by chest and central CT using a dual slice, high‐speed NX/I scanner (GE Medical Systems, Waukesha, WI), as previously described [21]. Scans were obtained using the axial, two‐slice scan mode (2i) and a segmented (a.k.a “half‐scan”) reconstruction to provide an effective temporal resolution of approximately 350 ms for each 3‐mm‐thick slice without cardiac gating. CAC values were obtained from cross‐sectional slices through the chest from the carina through the entire inferior aspect of the heart and measurements made by vessel for each of the major epicardial coronary arteries. For the abdominal scan series, a helical scan mode (120 KVp, 250 mA, 3‐mm slice thickness and pitch of 1.5:1) was utilized since the higher temporal resolution for the coronary arteries was not required. AAC values were obtained from cross‐sectional slices through the abdomen from L3 to S1 and included the summation of calcification in the abdominal aorta and common iliac arteries. Measurements were performed by experienced analysts using an FDA‐approved computer workstation and software (Calcium, Aquarius workstation, TeraRecon San Mateo, CA) that accounts for slice thickness and scan field of view [22]. The Agatston method [23] was used to report scores of calcified plaque. Using these methods, we have previously shown interclass correlation coefficient of > 0.99 for both CAC and AAC in 156 Coronary Artery Risk Development in Young Adults (CARDIA) Study scans that were blinded and re‐read [24]. Calcification was also modeled as a binary outcome, corresponding to the presence or absence of any calcified plaque as designated by an Agatston score > 10 versus not, respectively.

### 2.3. Olink Protein Panel

Blood samples were obtained by venipuncture after a 12‐h fast to prevent lipemia at the same clinic visit where markers of early atherosclerosis were measured. Specimens were collected in the morning to limit the effects of diurnal variation. For serum collection, blood tubes were allowed to stand at room temperature for a maximum of 20 min to clot before centrifugation. Serum was then aliquoted into 0.5‐mL cryovials and immediately frozen at −80°C in Tobago. Biospecimens were regularly shipped overnight on dry ice from Tobago to the University of Pittsburgh for long‐term storage.

Peripheral blood proteomic profiles were assayed at the University of Minnesota using the Olink Target 96 Cardiovascular III panel, a platform that quantifies the concentrations of 92 individual protein products that have been shown to be associated with CVD in previous research. The Olink platform is based on proximity extension assay (PEA) technology, which uses nucleotide‐labeled antibodies to bind to targeted proteins present in a sample and quantify their concentration via qPCR [25]. Quality control was performed using one intraplate control per plate and two interplate controls; calculated cross‐validation values averaged 5% for both sets of controls (range of 1%–14% for intraplate controls, 2%–19% for interplate controls).

### 2.4. Covariates

The ages of study participants at the time of sample collection were calculated using verified dates of birth. Trained staff conducted clinical exams to collect body mass index (BMI) and blood pressure data. BMI was calculated as body weight in kilograms divided by height in meters squared (from an average of two measurements). Systolic and diastolic blood pressures were calculated as the average of three measurements. Study staff also administered health history questionnaires which included questions about current/former smoking and previous diagnoses of diabetes mellitus. Stage 1 hypertension was defined according to the 2017 AHA guidelines as systolic BP ≥ 130, diastolic BP > 80, or the use of relevant medication and Stage 2 hypertension as systolic BP ≥ 140, diastolic BP ≥ 90. Stage 1 hypertension was used in statistical analyses due to its established relevance to cardiovascular health. Participants were classified as having diabetes by either a measure of fasting glucose ≥ 126 mg/dL or the use of relevant medication. Heart rate is automatically collected by the waveform analyzer that measures PWV. Low‐density lipoprotein (LDL) cholesterol was assessed via lipid profiling of blood samples from a previous study visit, according to a standard protocol.

### 2.5. Statistical Analysis

In addition to descriptive statistics, adjusted Spearman correlations were generated to analyze associations between protein markers and covariates (age, BMI, smoking status, diabetes, LDL cholesterol, and blood pressures). Correlations were adjusted for assay batch and age, except for correlations with age, which were only adjusted for assay batch. The relationship between protein concentrations and measures of early atherosclerosis was analyzed via differential expression analysis. Differential expression analysis is a method for analyzing high‐dimensional data collected from large‐scale molecular biology assays with the goal of identifying markers (genes, proteins, etc.) that are differentially expressed with respect to the presence or absence (or high or low value) of an outcome condition. The approach involves using moderated t‐statistics that are calculated using an empirical Bayes approach to shrink sample variances toward a pooled estimator. This method improves statistical power to detect differential expression and prevents highly variable proteins from skewing results [26]. Initial models were adjusted for assay batch, and subsequent models were additionally adjusted for age, BMI, smoking status, diabetes, LDL cholesterol, and blood pressures. Heart rate was also included in the fully adjusted model for PWV only. Diastolic blood pressure was residualized against systolic blood pressure before inclusion in models to prevent multicollinearity. AAC and CAC Agatston scores were transformed by adding 1 and taking the natural log due to the scores being non‐normally distributed. The Benjamini–Hochberg false discovery rate calculation was used to account for multiple testing. Differential expression analysis was performed using the Linear Models for Microarray Data (LIMMA) package [27] in *R* Statistical Software (v4.3.1; *R* Core Team 2023).

## 3. Results

### 3.1. Study Population

Characteristics of the study population are shown in Table 1. The average age of the study participants was 63.4 ± 8.1 years, and average BMI was 27.7 ± 4.3 kg/m^2^, corresponding to an overweight BMI. Nearly 80% of the study population was Stage 1 hypertensive, with an average systolic blood pressure of 142.9 ± 21.8 mmHg, while 24.6% had a diagnosis of diabetes. Average LDL cholesterol concentration was 129.4 ± 37.7 mg/dL. AAC was detected in 65.5% of the population, while CAC was present in 34.2%. Average PWV was 1594.5 m/s (IQR: 1402–1844).

**TABLE 1:** Analytic sample characteristics (*N* = 342).

Variable		Mean ± SD, median [IQR], or %
Age		63.3 ± 8.1 years
BMI		27.7 ± 4.3 kg/m2

Hypertension	Stage 1 or 2	79.2%
	Stage 2	63.6%

Systolic BP		142.9 ± 21.8 mmHg

Diastolic BP		79.8 ± 12.0 mmHg

LDL cholesterol		129.4 ± 37.7 mg/dL

Diabetes		24.6%

Current smoker		5.9%

PWV		1594.5 [1402, 1844] cm/s

AAC presence		60.8%

CAC presence		34.2%

Transformed AAC score		4.6 [0, 6.5]

Transformed CAC score		0 [0, 3.4]

*Note:* BMI = body mass index (weight in kg/height in meters [2]). Hypertension was defined according to the 2017 AHA guidelines (> 80 mmHg diastolic or ≥ 130 mmHg systolic or use of relevant medication for Stage 1; ≥ 90 mmHg diastolic or ≥ 140 mmHg systolic for Stage 2). AAC and CAC Agatston scores transformed by adding one and taking natural log.

Abbreviations: AAC = abdominal aortic calcification, CAC = coronary artery calcification, PWV = pulse wave velocity.

### 3.2. Adjusted Spearman Correlations

The results of adjusted Spearman correlations are presented in Supporting Table 2. After adjusting for assay batch, 39 markers were significantly associated with age. In assay‐batch‐ and age‐adjusted correlations, 74 of the 92 markers in the panel were associated with at least one CVD risk factor (BMI, smoking, diabetes, LDL cholesterol, systolic blood pressure, and diastolic blood pressure). The most significant associations were observed for BMI (26), followed by systolic blood pressure (21). Diastolic blood pressure, diabetes status, and smoking status were associated with 14 markers each, and LDL cholesterol was associated with 12. There was substantial heterogeneity in correlations with different covariates, and none of the markers were associated with all of them.

### 3.3. LIMMA Models

**FIGURE 1:**
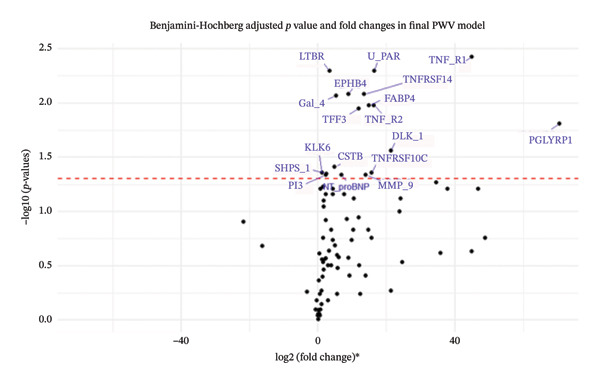
Plot of protein associations with PWV in fully adjusted models. Markers labeled in blue are significant in final PWV model after Benjamini–Hochberg FDR correction. Dashed red line corresponds to the *p* value of 0.05 (−log10(0.05) ≈ 1.30).

The results of linear models are shown in Table 2 and in Supporting Table 3. In initial models (adjusted for assay batch only) 43 individual protein markers were differentially expressed with respect to PWV values. Of these, 32 remained significant after adjusting for age, and 18 were significant after additionally adjusting for BMI, smoking, diabetes, LDL cholesterol, blood pressure, and heart rate: tumor necrosis factor receptor superfamily 14 (TNFRSF14), tumor necrosis factor receptor superfamily 10c (TNFRSF10c), lymphotoxin beta receptor (LTBR), galectin 4 (GAL4), ephrin type‐B receptor 4 (EPHB4), trefoil factor 3 (TFF3), peptidoglycan recognition protein 1 (PGLYRP1), protein delta homolog 1 (DLK1), tyrosine phosphatase substrate 1 (SHPS1), kallikrein‐6 (KLK6), peptidase inhibitor 3 (PI3), cystatin B (CSTB), urokinase receptor (UPAR), TNFR1 and TNFR2, fatty acid binding protein 4 (FABP4), NTproBNP, and MMP9 (Table 2, Figure 1, Supporting Table 3). In the assay batch–adjusted model, 23 and 22 protein markers were associated with transformed AAC and CAC Agatston scores, respectively. After adjusting for age, most of the relationships with calcification outcomes were attenuated, resulting in 4 markers associated with CAC scores—CSTB, FABP4, carboxypeptidase A1 (CPA1), and GAL4—and only 1 associated with AAC (CSTB). In the fully adjusted model, all associations with AAC were attenuated; only CSTB remained associated with CAC (Table 2, Supporting Table 3).

**TABLE 2:** Number of significant protein associations with markers of atherosclerosis in linear models.

CVD outcome measure	Model 1	Model 2	Full model
Pulse wave velocity	43	32	18
CAC presence	13	0	0
CAC score	22	4	1
AAC presence	0	0	0
AAC score	23	1	0

*Note:* Model 1 adjusted for assay batch. Model 2 additionally adjusted for age. Full model additionally adjusted for BMI, smoking status, diabetes diagnosis, LDL cholesterol, and systolic and diastolic blood pressure; PWV outcome also adjusted for heart rate.

## 4. Discussion

After adjustment for traditional CVD risk factors, we found that 18 protein biomarkers were associated with PWV and 1 was associated with CAC in a unique, Afro‐Caribbean population. Our study is the first to report the results of a large‐scale protein assay in an Afro‐Caribbean population, and 12 of the 18 protein associations with PWV appear to be novel: TNFRSF14, TNFRSF10c, LTBR, GAL4, EPHB4, TFF3, PGLYRP1, DLK1, SHPS1, KLK6, PI3, and CSTB. While CAC and AAC scores were associated with 22 and 23 biomarkers, respectively, in base models, most of these associations were attenuated by age adjustment, and only one (CSTB) remained significant with the CAC score after accounting for traditional CVD risk factors.

By design in using the Olink CVD III platform, all the proteins included in our analysis are known to be related to some component of CVD physiology. Our study is the first to establish the relevance of these markers in the Tobagonians by reporting significant age‐adjusted correlations with CVD risk factors that are known to be important in this population (Supporting Table 2). After adjustment, 74 protein markers were correlated with at least one covariate, with the highest number of associations observed for BMI (26) and systolic blood pressure (21). In differential expression analysis, adjusting for these covariates attenuated many of the relationships between protein markers and atherosclerosis markers, suggesting that part of the relationship between these markers and CVD progression is explained by biological pathways captured by traditional risk factors. By identifying a subset of proteins that are related to PWV, which is a sensitive measure of arterial stiffness and is related to vascular aging and a burden of hypertensive disease, our analysis contributes to the growing body of literature relating these markers to CVD processes. In addition, many of these associations were independent of traditional CVD risk factors, including age, BMI, smoking, diabetes, LDL cholesterol, and blood pressure. This suggests that these protein markers may provide additional information for predicting future risk of CVD that is not captured by traditional lifestyle and clinical risk factors.

Our analysis corroborates the results of previous studies that have reported associations between PWV and six of the markers that were identified in our analysis—UPAR [28], TNFR1 and TNFR2 [29], FABP4 [30], NTproBNP [31], and MMP9 [32]. One previous study that is particularly relevant to the current analysis investigated the relationship between the same Olink CVD III panel and PWV in a mixed‐race cohort in South Africa [30]. That study identified 7 protein makers associated with PWV (growth differentiation factor 15, E‐selectin, CPA1, FABP4, C‐X‐C motif chemokine 16, carboxypeptidase B, and tissue‐type plasminogen activator), including FABP4 which was also identified in the current study with similar directions of association. There may be many reasons for inconsistency between studies including power, differences in the specific outcomes assessed (i.e., brachial ankle versus cfPWV), and/or differences in the underlying populations tested. Importantly, previous studies have demonstrated that protein markers of CVD tend to differ between racial groups [14] and that, in general, protein biomarker analyses in African‐ancestry cohorts can reveal previously unidentified markers of CVD [12]. Our study supports these findings by reporting novel associations between protein markers and PWV in a previously unstudied and genetically unique population.

Our study also identified 12 novel proteins associated with PWV (Figure 1, Supporting Table 3). While the association with PWV specifically is novel, all of these have been shown in previous work to be related to various physiological mechanisms of CVD, which is expected because of the design of the Olink CVD III panel. Galectin 4 has previously been shown to be a predictor of coronary artery disease and stroke [33]. EPHB4 has primarily been studied in relation to vascular development and congenital heart disease [34] and has not been previously associated with CVD outcomes in adults. Cystatin B has been associated with PAD [35] but, unlike closely related cystatin C (which is not included in the CVD III panel), has not previously been associated with PWV [36] or CAC [37]. PGLYRP1 is an inflammatory protein that may attenuate atherosclerosis [38] and has previously been associated with CAC [39], but not PWV. TNFRSF14 and TNFRSF10c belong to the tumor necrosis factor superfamily. TNFRSF14 is related to immune signaling and inflammatory processes [40] and has previously been associated with cardiovascular events [41], while TNFRSF10c plays a role in vascular smooth muscle growth [42] and is a predictor of rehospitalization after heart failure [43]. LTBR, another member of the tumor necrosis factor superfamily, has been associated with coronary plaque [44]. TFF3 has been associated with atherosclerosis and peripheral artery disease [45]. KLK6 has been associated with cardiorespiratory fitness [46]. Although PI3 is a novel marker of arterial stiffness, the closely related PI4 has been studied as a potential therapeutic to reduce PWV [47]. Mouse models have provided evidence that DLK1 [48] and SHPS1 [49] may be related to the progression of CVD, but to the best of our knowledge, neither has been shown to be associated with CVD in humans. Thus, our study supports existing literature about these proteins by reporting an association between them and PWV, which has not been observed previously.

The associations between three of the four protein markers associated with CAC in age‐adjusted models and 14 of the proteins associated with PWV in age‐adjusted models were attenuated by further adjusting for traditional risk factors (Supporting Table 3). This indicates that these proteins may be markers of accelerated vascular aging and calcification but do not provide additional prognostic information that is not already captured by traditional risk factors.

It is important to note several limitations of our study. Due to the design of the THS and samples available at the time of Olink assay, our analysis is conducted in men only. However, we have recent evidence that the burden of risk factors differs substantially between Tobagonian men and women [50], which could mean that the current results may not necessarily be applicable to Tobagonian women. In addition, proteomic profiling was performed at a single time point, providing a snapshot of current protein concentrations but preventing us from analyzing how changes in protein concentrations over time may be related to CVD progression. The panel used to quantify protein concentrations only provides data on 92 preselected proteins, which limits our analysis from revealing any untested novel protein markers that predict early atherosclerosis progression in Afro‐Caribbeans. Lastly, the current study sample was relatively small, especially given the large number of predictors being analyzed, the false discovery rate correction used in our analysis, and adjustment for several covariates. As such, we may have been particularly limited in the analysis of vascular calcification outcomes which have less variability in the THS. However, we hope to replicate and extend these findings in future work, including increasing the sample size of men and including women who have recently been recruited in the THS and using an unbiased, proteomic approach. Nonetheless, even with these noted limitations, in a sample of Afro‐Caribbean men, we identified 18 proteins significantly and independently associated with PWV and of which, 12 appear to represent novel findings from human research.

The current study also has several strengths. Our work represents the first large‐scale protein quantification study of CVD in an Afro‐Caribbean population. The THS was drawn from a population of Afro‐Caribbean adults living in a relatively rural and economically underdeveloped region who are known to have a very high burden of CVD risk factors [15–17]. The majority of existing CVD research has focused on European‐ancestry populations, and even research in African‐ancestry groups tends to focus on African‐American populations that are genetically and culturally distinct from Afro‐Caribbeans [51]. As such, further research will be needed to comprehensively assess the generalizability of our results; however, the idea that the relationship between certain protein markers and CVD may be population‐specific is still clinically important and highlights the need for further work in precision medicine approaches. In addition, we used highly sensitive and predictive markers of atherosclerosis progression, specifically PWV and vascular calcification assessment. These measures allow us to gain a deeper understanding of the development of CVD throughout life before overt disease is apparent [1, 2, 52, 53]. Importantly, PWV and vascular calcification represent different mechanisms by which CVD develops and may reflect overlapping but distinct biological pathways; using both outcomes provides greater information than would be provided by either measure alone. The sensitivity of these measures also provides greater statistical power to detect differences in protein effects. Also, the Olink platform is a valid and reproducible method for quantifying protein concentrations in biological samples [54].

Profiling peripheral blood protein concentrations may be a useful method for assessing the risk of future CVD among Afro‐Caribbean men. In particular, the protein/CVD associations that remained significant after adjusting for traditional CVD risk factors have the potential to improve risk stratification in clinical settings by providing additional information that is not captured by commonly used methods for assessing risk. Further research is needed to fully understand the unique progression of CVD among this high‐risk group and the role that various proteins may play as clinical biomarkers of disease risk and to highlight potential mechanistic pathways of disease. A deeper understanding of the biological pathways involved in the progression of CVD throughout life will allow researchers and clinicians to identify individuals at high risk for future disease and tailor interventions that can slow or prevent the development of CVD.

^∗^Log2(fold change) values were truncated at 75.

## Funding

This study was funded by the National Heart, Lung, and Blood Institute, R01HL143793, K01HL125658, and R03HL144668; National Institute of Diabetes and Digestive and Kidney Diseases, R01DK097084.

## Conflicts of Interest

The authors declare no conflicts of interest.

## Supporting Information

Additional supporting information can be found online in the Supporting Information section.

## Supporting information


**Supporting Information** Supporting 1. Supporting table 1 contains the results of a sensitivity analysis comparing descriptive statistics for the random sample selected to undergo protein concentration profiling and the rest of the study cohort that was not included in this analysis. Supporting 2. Supporting table 2 presents the results of adjusted Spearman correlations between each of the 92 markers in our panel and the covariates we adjusted for in our differential expression analysis. Supporting 3. Supporting table 3 contains detailed results from differential expression analysis; in particular, adjusted *p* values and standardized fold changes for every protein marker that was statistically significant in any differential expression model.

## Data Availability

The data that support the findings of this study are available from the corresponding author upon reasonable request.
