# Supplementary material for: Large‐Scale Protein Assay Identifies Novel Protein Biomarkers Associated With Arterial Stiffness and Vascular Calcification Measures
Source: Int J Hypertens. 2026 Jul 14;2026:9724102. doi: 10.1155/ijhy/9724102 (PMC13366776; doi:10.1155/ijhy/9724102)
Supplement: Supplementary file 1 — Supporting Information Supporting 1. Supporting table 1 contains the results of a sensitivity analysis comparing descriptive statistics for the random sample selected to undergo protein concentration profiling and the rest of the study cohort that was not included in this analysis. Supporting 2. Supporting table 2 presents the results of adjusted Spearman correlations between each of the 92 markers in our panel and the covariates we adjusted for in our differential expression analysis. Supporting 3. Supporting table 3 contains detailed results from differential expression analysis; in particular, adjusted p values and standardized fold changes for every protein marker that was statistically significant in any differential expression model. [file IJHY-2026-9724102-s001.docx]

| **Supplemental Table 1: Comparison of Analytic Sample Characteristics and Full Tobago Health Study** | | | | |  |
| --- | --- | --- | --- | --- | --- |
|  | | **Mean ± SD, Median [IQR], or %​** | |  | |
| **Variable** | | **Analytic sample (n = 342)** | **Rest of THS (n = 514)** | **Comparison**  **p-value** | |
| Age​ | | 63.3 ± 8.1 years​ | 64.8 ± 9.4 years​ | 0.02 | |
| BMI​ | | 27.6 ± 4.3 kg/m2​ | 27.6 ± 4.8 kg/m2​ | 0.90 | |
| Hypertension | Stage 1 or 2 | 79.2% | 75.9%​ | 0.25 | |
|  | Stage 2 | 63.6%​ | 58.3% | 0.17 | |
| Systolic BP​ | | 142.9 ± 21.8 mmHg​ | 141.9 ± 23.3 mmHg​ | 0.50 | |
| Diastolic BP​ | | 79.8 ± 12.0 mmHg​ | 79.2 ± 12.5 mmHg​ | 0.51 | |
| LDL cholesterol | | 129.4 ± 37.7 mg/dL | 127.3 ± 38.8 mg/dL | 0.39 | |
| Diabetes​ | | 24.6%​ | 22.9%​ | 0.58 | |
| Current smoker​ | | 5.9%​ | 9.4%​ | 0.06 | |
| PWV (cm/s)​ | | 1594.5 [1402.0, 1844.0]​ | 1554.1 [1377.0, 1791.1]​ | 0.22 | |
| AAC​ presence | | 60.8%​ | 64.6%​ | 0.29 | |
| CAC presence | | 28.7%​ | 28.2%​ | 0.88 | |
| Transformed AAC score | | 4.6 [0, 6.5] | 4.8 [0, 6.6] | 0.18 | |
| Transformed CAC score | | 0 [0, 3.4] | 0 [0, 3.2] | 0.79 | |

BMI = body mass index (weight in kg / height in meters^2^)

Hypertension was defined according to 2017 AHA guidelines (>80 mmHg diastolic or ≥130 mmHg systolic or use of relevant medication for stage 1; ≥90 mmHg diastolic or ≥140 mmHg systolic for stage 2)

PWV = pulse wave velocity (in m/s)

AAC = abdominal aortic calcification

CAC = coronary artery calcification

AAC and CAC scores transformed by adding one and taking natural log

Comparison p-values: T-test for continuous variables (Satterthwaite if equality of variances F-test p-value < 0.05; otherwise pooled variances), chi-squared test for categorical variables (Fisher’s exact test not needed as all expected cell frequencies are >5)

**Supplemental Table 2. Adjusted Spearman Correlations between Protein Markers and Covariates**

| **Protein name** | **Protein abbreviation** | **Protein symbol** | **Age** | | **LDL cholesterol** | | **Systolic BP** | | **Diastolic BP** | |
| --- | --- | --- | --- | --- | --- | --- | --- | --- | --- | --- |
|  |  |  | ***r*** | **p** | ***r*** | **p** | ***r*** | **p** | ***r*** | **p** |
| Tumor necrosis factor receptor superfamily member 14 | TNFRSF14 | Q92956 | -0.02 | 0.77 | -0.05 | 0.44 | 0.10 | 0.07 | 0.08 | 0.15 |
| Low-density lipoprotein receptor | LDLr | P01130 | **-0.13** | **0.02** | **0.17** | **0.003** | **0.17** | **0.001** | 0.18 | **0.0007** |
| Integrin beta-2 | ITGB2 | P05107 | -0.04 | 0.51 | 0.02 | 0.79 | 0.08 | 0.15 | 0.04 | 0.47 |
| Interleukin-17 receptor A | IL17RA | Q96F46 | -0.05 | 0.41 | 0.01 | 0.89 | -0.01 | 0.83 | -0.03 | 0.64 |
| Tumor necrosis factor receptor 2 | TNFR2 | P20333 | **0.20** | **0.0003** | -0.08 | 0.20 | 0.06 | 0.26 | 0.01 | 0.92 |
| Matrix metallopeptidase 9 | MMP9 | P14780 | -0.01 | 0.84 | 0.00 | 0.98 | -0.02 | 0.70 | 0.03 | 0.58 |
| Ephrin type-B receptor 4 | EPHB4 | P54760 | **0.17** | **0.002** | -0.011 | 0.06 | 0.03 | 0.56 | 0.00 | 0.99 |
| Interleukin-2 receptor alpha chain | IL2RA | P01589 | **0.15** | **0.01** | **-0.12** | **0.04** | 0.01 | 0.85 | -0.06 | 0.27 |
| Osteoprotegerin | OPG | O00300 | **0.37** | **<0.0001** | 0.00 | 0.99 | 0.16 | 0.002 | 0.09 | 0.10 |
| Activated leukocyte cell adhesion molecule | ALCAM | Q13740 | 0.08 | 0.14 | -0.04 | 0.54 | 0.09 | 0.08 | 0.06 | 0.29 |
| Trefoil factor 3 | TFF3 | Q07654 | **0.30** | **<0.0001** | -0.06 | 0.33 | 0.00 | 1.00 | -0.01 | 0.87 |
| P-selectin | SELP | P16109 | -0.07 | 0.20 | **0.12** | **0.04** | 0.03 | 0.57 | 0.07 | 0.21 |
| Cystatin B | CSTB | P04080 | **0.12** | **0.03** | -0.03 | 0.67 | 0.09 | 0.10 | 0.10 | 0.08 |
| Monocyte chemoattractant protein-1 | MCP1 | P13500 | 0.10 | 0.06 | -0.07 | 0.23 | 0.01 | 0.92 | 0.00 | 0.94 |
| Cluster of Differentiation 163 | CD163 | Q86VB7 | 0.05 | 0.36 | -0.06 | 0.35 | 0.10 | 0.06 | 0.06 | 0.25 |
| Galectin-3 | Gal3 | P17931 | **0.18** | **0.001** | 0.05 | 0.44 | 0.07 | 0.19 | 0.08 | 0.16 |
| Granulin precursor | GRN | P28799 | 0.00 | 0.99 | 0.02 | 0.69 | 0.06 | 0.23 | 0.06 | 0.29 |
| N-terminal pro b-type natriuretic peptide | NTproBNP | P16860 | **0.36** | **<0.0001** | -0.11 | 0.07 | **0.11** | **0.04** | 0.04 | 0.46 |
| Bloom syndrome protein homolog | BLMh | Q13867 | -0.03 | 0.65 | 0.04 | 0.55 | 0.10 | 0.07 | 0.06 | 0.28 |
| Phospholipase C | PLC | P98160 | **0.14** | **0.01** | -0.03 | 0.67 | **0.14** | **0.01** | 0.10 | 0.06 |
| Lymphotoxin beta receptor | LTBR | P36941 | **0.19** | **0.0007** | -0.08 | 0.16 | 0.09 | 0.11 | 0.03 | 0.57 |
| Neurogenic locus notch homolog protein 3 | Notch3 | Q9UM47 | **0.20** | **0.0004** | -0.05 | 0.42 | 0.11 | 0.048 | 0.06 | 0.29 |
| Tissue inhibitor of metalloproteinases 4 | TIMP4 | Q99727 | **0.33** | **<0.0001** | -0.02 | 0.71 | 0.11 | 0.05 | 0.07 | 0.20 |
| Contactin-1 | CNTN1 | Q12860 | 0.11 | 0.05 | 0.04 | 0.50 | **0.11** | **0.04** | 0.09 | 0.10 |
| Cadherin-5 | CDH5 | P33151 | 0.01 | 0.87 | -0.04 | 0.47 | 0.01 | 0.81 | 0.01 | 0.93 |
| Triggering receptor expressed on myeloid cells-like transcript 2 | TLT2 | Q5T2D2 | -0.04 | 0.49 | -0.04 | 0.54 | 0.10 | 0.05 | 0.07 | 0.18 |
| Fatty acid-binding protein 4 | FABP4 | P15090 | **0.14** | **0.01** | 0.11 | 0.07 | **0.17** | **0.002** | **0.20** | **0.0002** |
| Tissue factor pathway inhibitor | TFPI | P10646 | 0.00 | 0.94 | **0.22** | **0.0001** | 0.09 | 0.09 | **0.13** | **0.02** |
| Plasminogen activator inhibitor | PAI | P05121 | **-0.15** | **0.01** | 0.01 | 0.87 | **0.11** | **0.04** | **0.15** | **0.01** |
| C-C motif chemokine ligand 24 | CCL24 | O00175 | -0.04 | 0.53 | 0.09 | 0.15 | 0.01 | 0.90 | 0.06 | 0.27 |
| Thyroid hormone receptor | TR | P02786 | 0.05 | 0.34 | 0.09 | 0.12 | 0.04 | 0.48 | -0.04 | 0.42 |
| Tumor necrosis factor receptor superfamily member 10C | TNFRSF10C | O14798 | 0.08 | 0.14 | -0.10 | 0.10 | 0.00 | 0.95 | -0.02 | 0.77 |
| Growth/differentiation factor 15 | GDF15 | Q99988 | **0.48** | **<0.0001** | -0.10 | 0.10 | 0.03 | 0.64 | -0.01 | 0.84 |
| E-selectin | SELE | P16581 | **-0.15** | **0.01** | 0.05 | 0.45 | 0.10 | 0.06 | **0.12** | **0.03** |
| Azurocidin 1 | AZU1 | P20160 | -0.02 | 0.76 | 0.02 | 0.70 | -0.02 | 0.72 | 0.01 | 0.85 |
| Delta-like 1 homolog | DLK1 | P80370 | **0.14** | **0.01** | 0.01 | 0.84 | **0.13** | **0.02** | 0.09 | 0.11 |
| Spondin-1 | SPON1 | Q9HCB6 | **0.21** | **0.0002** | -0.01 | 0.91 | **0.12** | **0.03** | 0.07 | 0.17 |
| Myeloperoxidase | MPO | P05164 | -0.02 | 0.73 | -0.02 | 0.71 | -0.02 | 0.68 | 0.01 | 0.91 |
| C-X-C motif chemokine ligand 16 | CXCL16 | Q9H2A7 | 0.05 | 0.35 | 0.00 | 0.99 | 0.06 | 0.28 | 0.04 | 0.47 |
| Interleukin-6 receptor alpha chain | IL6RA | P08887 | 0.00 | 0.97 | -0.02 | 0.76 | 0.05 | 0.32 | 0.03 | 0.57 |
| Resistin | RETN | Q9HD89 | 0.05 | 0.42 | -0.01 | 0.84 | -0.02 | 0.69 | -0.03 | 0.64 |
| Insulin-like growth factor-binding protein 1 | IGFBP1 | P08833 | **0.33** | **<0.0001** | **-0.13** | **0.03** | **-0.20** | **0.0002** | **-0.22** | **<0.0001** |
| Chitotriosidase-1 | CHIT1 | Q13231 | **0.13** | **0.02** | 0.06 | 0.32 | 0.01 | 0.86 | -0.01 | 0.80 |
| Tartrate-resistant acid phosphatase | TRAP | P13686 | 0.01 | 0.90 | 0.02 | 0.70 | **0.15** | **0.01** | **0.13** | **0.01** |
| Platelet glycoprotein VI | GP6 | Q9HCN6 | **-0.12** | **0.03** | 0.03 | 0.65 | 0.03 | 0.56 | 0.05 | 0.32 |
| Pulmonary surfactant-associated protein D | PSPD | P35247 | -0.06 | 0.27 | -0.06 | 0.34 | 0.08 | 0.15 | 0.06 | 0.24 |
| Peptidase inhibitor 3 | PI3 | P19957 | **0.25** | **<0.0001** | -0.04 | 0.54 | 0.06 | 0.26 | 0.00 | 0.97 |
| Epithelial cell adhesion molecule | EpCAM | P16422 | 0.05 | 0.34 | -0.05 | 0.37 | 0.02 | 0.77 | 0.00 | 0.98 |
| Aminopeptidase N | APN | P15144 | -0.01 | 0.89 | -0.01 | 0.89 | 0.10 | 0.06 | 0.10 | 0.07 |
| AXL receptor tyrosine kinase | AXL | P30530 | 0.01 | 0.91 | -0.09 | 0.13 | 0.09 | 0.11 | 0.03 | 0.60 |
| Interleukin-1 receptor type 1 | IL1RT1 | P14778 | 0.11 | 0.05 | -0.09 | 0.12 | 0.07 | 0.18 | 0.02 | 0.70 |
| Matrix metallopeptidase 2 | MMP2 | P08253 | **0.13** | **0.02** | -0.05 | 0.37 | **0.14** | **0.01** | 0.07 | 0.18 |
| FAS cell surface death receptor | FAS | P25445 | 0.10 | 0.07 | -0.03 | 0.66 | 0.11 | 0.05 | 0.04 | 0.52 |
| Myoglobin | MB | P02144 | **0.13** | **0.02** | 0.02 | 0.72 | 0.06 | 0.25 | 0.04 | 0.46 |
| Tumor necrosis factor ligand superfamily member 13B | TNFSF13B | Q9Y275 | 0.09 | 0.12 | -0.03 | 0.60 | 0.04 | 0.49 | 0.01 | 0.85 |
| Proteinase 3 | PRTN3 | P24158 | -0.01 | 0.90 | 0.01 | 0.82 | 0.01 | 0.82 | 0.05 | 0.32 |
| Proprotein convertase subtilisin/kexin type 9 | PCSK9 | Q8NBP7 | 0.04 | 0.43 | **0.15** | **0.01** | 0.08 | 0.15 | **0.12** | **0.03** |
| Urokinase plasminogen activator surface receptor | UPAR | Q03405 | 0.09 | 0.12 | -0.02 | 0.78 | 0.06 | 0.31 | 0.07 | 0.22 |
| Osteopontin | OPN | P10451 | **0.16** | **0.01** | -0.10 | 0.11 | 0.06 | 0.24 | 0.00 | 0.96 |
| Cathepsin D | CTSD | P07339 | 0.02 | 0.78 | 0.03 | 0.67 | **0.14** | **0.01** | 0.10 | 0.06 |
| Peptidoglycan recognition protein 1 | PGLYRP1 | O75594 | -0.01 | 0.84 | -0.05 | 0.39 | 0.03 | 0.60 | 0.06 | 0.31 |
| Carboxypeptidase A1 | CPA1 | P15085 | -0.02 | 0.78 | -0.02 | 0.68 | 0.04 | 0.49 | 0.10 | 0.08 |
| Junctional adhesion molecule A | JAMA | Q9Y624 | -0.11 | 0.05 | 0.00 | 0.94 | 0.03 | 0.63 | 0.05 | 0.41 |
| Galectin-4 | Gal4 | P56470 | **0.26** | **<0.0001** | -0.08 | 0.16 | -0.04 | 0.50 | -0.05 | 0.35 |
| Interleukin-1 receptor type 2 | IL1RT2 | P27930 | -0.09 | 0.12 | -0.03 | 0.66 | 0.08 | 0.15 | 0.07 | 0.21 |
| Src homology 2 domain-containing protein tyrosine phosphatase substrate 1 | SHPS1 | P78324 | 0.07 | 0.21 | **-0.15** | **0.01** | 0.07 | 0.22 | -0.02 | 0.68 |
| C-C motif chemokine ligand 15 | CCL15 | Q16663 | **0.13** | **0.02** | 0.03 | 0.56 | 0.08 | 0.16 | 0.01 | 0.90 |
| Caspase-3 | CASP3 | P42574 | **-0.15** | **0.01** | 0.04 | 0.52 | 0.01 | 0.81 | 0.03 | 0.59 |
| Urokinase-type plasminogen activator | uPA | P00749 | 0.01 | 0.89 | -0.07 | 0.23 | 0.09 | 0.10 | 0.07 | 0.23 |
| Carboxypeptidase B1 | CPB1 | P15086 | 0.02 | 0.68 | 0.00 | 0.99 | 0.10 | 0.08 | **0.14** | **0.01** |
| Chitinase-3-like protein 1 | CHI3L1 | P36222 | **0.18** | **0.001** | **-0.13** | **0.02** | 0.10 | 0.06 | 0.04 | 0.47 |
| Interleukin-1 receptor-like 1 | ST2 | Q01638 | **0.12** | **0.03** | -0.03 | 0.58 | 0.04 | 0.49 | 0.01 | 0.92 |
| Tissue-type plasminogen activator | tPA | P00750 | 0.00 | 0.98 | **0.16** | **0.001** | **0.28** | **<0.0001** | **0.30** | **<0.0001** |
| Secretoglobin family 3A member 2 | SCGB3A2 | Q96PL1 | **0.12** | **0.02** | -0.05 | 0.37 | -0.09 | 0.09 | -0.09 | 0.11 |
| Epidermal growth factor receptor | EGFR | P00533 | **-0.11** | **0.04** | 0.07 | 0.25 | **0.14** | **0.01** | **0.14** | **0.01** |
| Insulin-like growth factor-binding protein 7 | IGFBP7 | Q16270 | **0.13** | **0.02** | -0.04 | 0.47 | **0.13** | **0.01** | 0.09 | 0.10 |
| Cluster of differentiation 93 | CD93 | Q9NPY3 | 0.05 | 0.34 | **-0.14** | **0.02** | 0.03 | 0.58 | -0.05 | 0.40 |
| Interleukin-18 binding protein | IL18BP | O95998 | **0.18** | **0.001** | -0.08 | 0.20 | 0.05 | 0.31 | 0.00 | 0.94 |
| Collagen type I alpha 1 chain | COL1A1 | P02452 | 0.05 | 0.39 | **-0.14** | **0.02** | 0.05 | 0.39 | 0.02 | 0.75 |
| Paraoxonase 3 | PON3 | Q15166 | -0.05 | 0.39 | -0.08 | 0.21 | 0.07 | 0.23 | 0.08 | 0.13 |
| Cathepsin Z | CTSZ | Q9UBR2 | 0.10 | 0.09 | -0.08 | 0.18 | 0.10 | 0.07 | 0.05 | 0.32 |
| Matrix metallopeptidase 3 | MMP3 | P08254 | 0.03 | 0.60 | -0.01 | 0.82 | **0.12** | **0.03** | 0.05 | 0.31 |
| Retinoic acid receptor responder 2 | RARRES2 | Q99969 | 0.02 | 0.66 | 0.08 | 0.18 | **0.12** | **0.03** | **0.13** | **0.02** |
| Intercellular adhesion molecule 2 | ICAM2 | P13598 | 0.02 | 0.69 | -0.05 | 0.42 | 0.03 | 0.63 | 0.06 | 0.25 |
| Kallikrein-related peptidase 6 | KLK6 | Q92876 | 0.10 | 0.07 | -0.05 | 0.41 | 0.07 | 0.20 | 0.04 | 0.44 |
| Platelet-derived growth factor subunit A | PDGFsubA | P04085 | **-0.14** | **0.01** | -0.03 | 0.58 | 0.08 | 0.16 | 0.11 | 0.04 |
| Tumor necrosis factor receptor 1 | TNFR1 | P19438 | **0.20** | **0.0004** | -0.09 | 0.13 | 0.06 | 0.24 | 0.03 | 0.60 |
| Insulin-like growth factor-binding protein 2 | IGFBP2 | P18065 | **0.35** | **<0.0001** | **-0.20** | **0.0009** | **-0.18** | **0.0006** | **-0.21** | **<0.0001** |
| Von Willebrand factor | vWF | P04275 | **0.22** | **<0.0001** | 0.05 | 0.40 | -0.01 | 0.79 | -0.02 | 0.68 |
| Platelet and endothelial cell adhesion molecule 1 | PECAM1 | P16284 | -0.10 | 0.08 | -0.02 | 0.80 | 0.04 | 0.42 | 0.05 | 0.41 |
| Matrix extracellular phosphoglycoprotein | MEPE | Q9NQ76 | -0.04 | 0.51 | -0.07 | 0.27 | 0.05 | 0.34 | 0.04 | 0.45 |
| C-C motif chemokine ligand 16 | CCL16 | O15467 | 0.01 | 0.83 | 0.05 | 0.44 | **0.11** | **0.04** | 0.08 | 0.12 |

Spearman correlations were used because markers are non-normally distributed

All correlations were adjusted for assay batch; all correlations except those with age were adjusted for age

Significant associations (<0.05) are in bold

**Supplemental Table 2. Adjusted Spearman Correlations between Protein Markers and Covariates (cont.)**

| **Protein name** | **Protein abbreviation** | **Protein symbol** | **Diabetes** | | **BMI** | | **Smoking** | |
| --- | --- | --- | --- | --- | --- | --- | --- | --- |
|  |  |  | ***r*** | **p** | ***r*** | **p** | ***r*** | **p** |
| Tumor necrosis factor receptor superfamily member 14 | TNFRSF14 | Q92956 | 0.06 | 0.26 | 0.08 | 0.15 | 0.09 | 0.11 |
| Low-density lipoprotein receptor | LDLr | P01130 | **0.13** | **0.02** | **0.26** | **<0.0001** | 0.04 | 0.45 |
| Integrin beta-2 | ITGB2 | P05107 | **0.23** | **<0.0001** | 0.08 | 0.13 | -0.01 | 0.84 |
| Interleukin-17 receptor A | IL17RA | Q96F46 | 0.07 | 0.19 | -0.03 | 0.53 | -0.02 | 0.78 |
| Tumor necrosis factor receptor 2 | TNFR2 | P20333 | **0.11** | **0.03** | 0.06 | 0.24 | **0.11** | **0.04** |
| Matrix metallopeptidase 9 | MMP9 | P14780 | **0.22** | **<0.0001** | **0.14** | **0.01** | 0.07 | 0.19 |
| Ephrin type-B receptor 4 | EPHB4 | P54760 | 0.03 | 0.62 | -0.01 | 0.89 | 0.06 | 0.29 |
| Interleukin-2 receptor alpha chain | IL2RA | P01589 | -0.04 | 0.47 | -0.04 | 0.45 | **0.12** | **0.03** |
| Osteoprotegerin | OPG | O00300 | **0.14** | **0.01** | 0.05 | 0.34 | -0.01 | 0.83 |
| Activated leukocyte cell adhesion molecule | ALCAM | Q13740 | 0.10 | 0.06 | 0.01 | 0.81 | 0.07 | 0.19 |
| Trefoil factor 3 | TFF3 | Q07654 | 0.08 | 0.14 | -0.02 | 0.69 | 0.08 | 0.15 |
| P-selectin | SELP | P16109 | 0.05 | 0.37 | 0.07 | 0.21 | 0.07 | 0.19 |
| Cystatin B | CSTB | P04080 | 0.10 | 0.07 | **0.14** | **0.01** | **0.14** | **0.01** |
| Monocyte chemoattractant protein-1 | MCP1 | P13500 | 0.04 | 0.49 | -0.05 | 0.35 | **0.11** | **0.04** |
| Cluster of Differentiation 163 | CD163 | Q86VB7 | **0.15** | **0.004** | **0.15** | **0.01** | -0.03 | 0.64 |
| Galectin-3 | Gal3 | P17931 | 0.08 | 0.16 | 0.05 | 0.35 | 0.09 | 0.12 |
| Granulin precursor | GRN | P28799 | 0.10 | 0.08 | 0.04 | 0.44 | 0.07 | 0.23 |
| N-terminal pro b-type natriuretic peptide | NTproBNP | P16860 | 0.00 | 0.99 | -0.09 | 0.11 | 0.05 | 0.36 |
| Bloom syndrome protein homolog | BLMh | Q13867 | 0.00 | 0.93 | 0.02 | 0.67 | 0.02 | 0.77 |
| Phospholipase C | PLC | P98160 | -0.01 | 0.89 | **0.13** | **0.01** | 0.07 | 0.20 |
| Lymphotoxin beta receptor | LTBR | P36941 | 0.01 | 0.80 | 0.01 | 0.78 | 0.07 | 0.19 |
| Neurogenic locus notch homolog protein 3 | Notch3 | Q9UM47 | -0.02 | 0.68 | -0.06 | 0.27 | 0.04 | 0.46 |
| Tissue inhibitor of metalloproteinases 4 | TIMP4 | Q99727 | -0.01 | 0.80 | 0.09 | 0.09 | 0.00 | 0.94 |
| Contactin-1 | CNTN1 | Q12860 | **0.12** | **0.02** | -0.01 | 0.82 | 0.01 | 0.93 |
| Cadherin-5 | CDH5 | P33151 | 0.05 | 0.36 | -0.03 | 0.59 | 0.01 | 0.89 |
| Triggering receptor expressed on myeloid cells-like transcript 2 | TLT2 | Q5T2D2 | 0.05 | 0.33 | **0.11** | **0.04** | 0.07 | 0.18 |
| Fatty acid-binding protein 4 | FABP4 | P15090 | **0.23** | **<0.0001** | 0.56 | <0.0001 | 0.09 | 0.10 |
| Tissue factor pathway inhibitor | TFPI | P10646 | 0.03 | 0.59 | 0.02 | 0.73 | 0.09 | 0.11 |
| Plasminogen activator inhibitor | PAI | P05121 | 0.09 | 0.09 | **0.16** | **0.003** | 0.05 | 0.34 |
| C-C motif chemokine ligand 24 | CCL24 | O00175 | -0.05 | 0.39 | 0.04 | 0.41 | 0.04 | 0.42 |
| Thyroid hormone receptor | TR | P02786 | **0.14** | **0.01** | **0.11** | **0.04** | -0.05 | 0.36 |
| Tumor necrosis factor receptor superfamily member 10C | TNFRSF10C | O14798 | **0.12** | **0.03** | 0.04 | 0.47 | 0.09 | 0.11 |
| Growth/differentiation factor 15 | GDF15 | Q99988 | **0.28** | **<0.0001** | 0.06 | 0.30 | 0.10 | 0.07 |
| E-selectin | SELE | P16581 | **0.23** | **<0.0001** | **0.20** | **0.0003** | 0.08 | 0.13 |
| Azurocidin 1 | AZU1 | P20160 | **0.17** | **0.001** | **0.11** | **0.05** | 0.06 | 0.27 |
| Delta-like 1 homolog | DLK1 | P80370 | 0.00 | 0.97 | **0.18** | **0.001** | **0.11** | **0.04** |
| Spondin-1 | SPON1 | Q9HCB6 | 0.07 | 0.21 | 0.02 | 0.65 | 0.09 | 0.10 |
| Myeloperoxidase | MPO | P05164 | **0.18** | **0.001** | 0.08 | 0.16 | 0.06 | 0.28 |
| C-X-C motif chemokine ligand 16 | CXCL16 | Q9H2A7 | 0.08 | 0.12 | 0.09 | 0.11 | 0.10 | 0.05 |
| Interleukin-6 receptor alpha chain | IL6RA | P08887 | 0.06 | 0.26 | 0.00 | 0.98 | 0.08 | 0.13 |
| Resistin | RETN | Q9HD89 | **0.15** | **0.01** | **0.13** | **0.02** | 0.06 | 0.30 |
| Insulin-like growth factor-binding protein 1 | IGFBP1 | P08833 | **0.12** | **0.03** | **-0.43** | **<0.0001** | -0.02 | 0.68 |
| Chitotriosidase-1 | CHIT1 | Q13231 | -0.02 | 0.68 | -0.08 | 0.14 | **0.17** | **0.002** |
| Tartrate-resistant acid phosphatase | TRAP | P13686 | **0.19** | **0.0006** | 0.04 | 0.49 | 0.06 | 0.26 |
| Platelet glycoprotein VI | GP6 | Q9HCN6 | 0.00 | 0.94 | 0.03 | 0.64 | 0.05 | 0.32 |
| Pulmonary surfactant-associated protein D | PSPD | P35247 | -0.05 | 0.39 | -0.09 | 0.12 | **0.15** | **0.01** |
| Peptidase inhibitor 3 | PI3 | P19957 | 0.00 | 0.94 | -0.05 | 0.39 | 0.04 | 0.47 |
| Epithelial cell adhesion molecule | EpCAM | P16422 | -0.10 | 0.06 | **-0.13** | **0.02** | 0.11 | 0.05 |
| Aminopeptidase N | APN | P15144 | 0.09 | 0.09 | -0.02 | 0.70 | 0.02 | 0.71 |
| AXL receptor tyrosine kinase | AXL | P30530 | 0.04 | 0.46 | 0.03 | 0.63 | 0.03 | 0.57 |
| Interleukin-1 receptor type 1 | IL1RT1 | P14778 | **0.17** | **0.002** | -0.02 | 0.72 | 0.04 | 0.47 |
| Matrix metallopeptidase 2 | MMP2 | P08253 | 0.00 | 0.98 | -0.04 | 0.48 | 0.01 | 0.86 |
| FAS cell surface death receptor | FAS | P25445 | 0.03 | 0.58 | 0.05 | 0.39 | 0.09 | 0.11 |
| Myoglobin | MB | P02144 | 0.08 | 0.14 | **0.20** | **0.003** | 0.02 | 0.70 |
| Tumor necrosis factor ligand superfamily member 13B | TNFSF13B | Q9Y275 | 0.01 | 0.79 | 0.07 | 0.21 | **0.14** | **0.01** |
| Proteinase 3 | PRTN3 | P24158 | **0.17** | **0.002** | 0.09 | 0.12 | 0.11 | 0.05 |
| Proprotein convertase subtilisin/kexin type 9 | PCSK9 | Q8NBP7 | 0.02 | 0.70 | 0.07 | 0.21 | 0.02 | 0.77 |
| Urokinase plasminogen activator surface receptor | UPAR | Q03405 | 0.19 | 0.006 | 0.08 | 0.12 | **0.11** | **0.04** |
| Osteopontin | OPN | P10451 | -0.05 | 0.32 | -0.05 | 0.36 | 0.03 | 0.63 |
| Cathepsin D | CTSD | P07339 | **0.27** | **<0.0001** | **0.17** | **0.001** | 0.11 | 0.05 |
| Peptidoglycan recognition protein 1 | PGLYRP1 | O75594 | **0.15** | **0.01** | 0.07 | 0.18 | 0.04 | 0.45 |
| Carboxypeptidase A1 | CPA1 | P15085 | -0.02 | 0.77 | 0.09 | 0.09 | **0.12** | **0.03** |
| Junctional adhesion molecule A | JAMA | Q9Y624 | 0.05 | 0.31 | 0.04 | 0.49 | 0.06 | 0.28 |
| Galectin-4 | Gal4 | P56470 | **0.30** | **<0.0001** | 0.05 | 0.40 | 0.05 | 0.32 |
| Interleukin-1 receptor type 2 | IL1RT2 | P27930 | 0.09 | 0.11 | 0.04 | 0.51 | 0.05 | 0.40 |
| Src homology 2 domain-containing protein tyrosine phosphatase substrate 1 | SHPS1 | P78324 | **0.13** | **0.02** | 0.00 | 0.95 | 0.03 | 0.57 |
| C-C motif chemokine ligand 15 | CCL15 | Q16663 | 0.10 | 0.07 | 0.03 | 0.59 | 0.05 | 0.31 |
| Caspase-3 | CASP3 | P42574 | -0.02 | 0.67 | 0.00 | 0.99 | 0.05 | 0.39 |
| Urokinase-type plasminogen activator | uPA | P00749 | 0.01 | 0.92 | -0.03 | 0.53 | 0.04 | 0.50 |
| Carboxypeptidase B1 | CPB1 | P15086 | -0.04 | 0.44 | **0.13** | **0.02** | 0.09 | 0.10 |
| Chitinase-3-like protein 1 | CHI3L1 | P36222 | **0.20** | **0.002** | **0.16** | **0.0003** | 0.07 | 0.22 |
| Interleukin-1 receptor-like 1 | ST2 | Q01638 | **0.11** | **0.04** | -0.01 | 0.82 | 0.04 | 0.52 |
| Tissue-type plasminogen activator | tPA | P00750 | **0.20** | **0.0002** | **0.43** | **<0.0001** | 0.08 | 0.16 |
| Secretoglobin family 3A member 2 | SCGB3A2 | Q96PL1 | **-0.11** | **0.049** | **-0.30** | **<0.0001** | 0.09 | 0.10 |
| Epidermal growth factor receptor | EGFR | P00533 | **0.13** | **0.01** | 0.05 | 0.37 | 0.06 | 0.28 |
| Insulin-like growth factor-binding protein 7 | IGFBP7 | Q16270 | 0.00 | 0.97 | 0.02 | 0.70 | 0.05 | 0.38 |
| Cluster of differentiation 93 | CD93 | Q9NPY3 | -0.02 | 0.65 | -0.08 | 0.13 | **0.11** | **0.04** |
| Interleukin-18 binding protein | IL18BP | O95998 | 0.06 | 0.26 | -0.04 | 0.46 | **0.11** | **0.04** |
| Collagen type I alpha 1 chain | COL1A1 | P02452 | -0.11 | 0.05 | -0.09 | 0.09 | 0.02 | 0.68 |
| Paraoxonase 3 | PON3 | Q15166 | -0.07 | 0.20 | **-0.28** | **<0.0001** | -0.02 | 0.78 |
| Cathepsin Z | CTSZ | Q9UBR2 | **0.17** | **0.002** | 0.10 | 0.08 | 0.04 | 0.47 |
| Matrix metallopeptidase 3 | MMP3 | P08254 | -0.08 | 0.15 | **-0.11** | **0.04** | 0.02 | 0.67 |
| Retinoic acid receptor responder 2 | RARRES2 | Q99969 | 0.06 | 0.25 | **0.23** | **<0.0001** | **0.11** | **0.047** |
| Intercellular adhesion molecule 2 | ICAM2 | P13598 | 0.07 | 0.17 | 0.00 | 1.00 | 0.03 | 0.55 |
| Kallikrein-related peptidase 6 | KLK6 | Q92876 | -0.03 | 0.60 | -0.13 | 0.02 | -0.01 | 0.86 |
| Platelet-derived growth factor subunit A | PDGFsubA | P04085 | 0.02 | 0.77 | 0.02 | 0.72 | 0.08 | 0.13 |
| Tumor necrosis factor receptor 1 | TNFR1 | P19438 | 0.10 | 0.06 | 0.10 | 0.07 | **0.12** | **0.03** |
| Insulin-like growth factor-binding protein 2 | IGFBP2 | P18065 | **-0.15** | **0.004** | **-0.46** | **<0.0001** | 0.02 | 0.69 |
| Von Willebrand factor | vWF | P04275 | 0.08 | 0.15 | 0.00 | 0.95 | 0.03 | 0.54 |
| Platelet and endothelial cell adhesion molecule 1 | PECAM1 | P16284 | 0.07 | 0.20 | 0.00 | 0.96 | 0.05 | 0.37 |
| Matrix extracellular phosphoglycoprotein | MEPE | Q9NQ76 | -0.07 | 0.22 | -0.01 | 0.79 | 0.02 | 0.68 |
| C-C motif chemokine ligand 16 | CCL16 | O15467 | 0.06 | 0.24 | **0.17** | **0.002** | 0.08 | 0.15 |

Spearman correlations were used because markers are non-normally distributed

All correlations were adjusted for assay batch; all correlations except those with age were adjusted for age

Significant associations (<0.05) are in bold

**Supplemental Table 3. P-values and standardized fold changes for all markers with adjusted p-value <0.05 in any model**

**3a. Pulse wave velocity**

| Protein name | Protein abbreviation | Protein symbol | Outcome | Model 1 log_2_ fold change | Model 1 p | Model 2 log_2_ fold change | Model 2 p | Full Model log_2_ fold change | Full Model p | Novel? |
| --- | --- | --- | --- | --- | --- | --- | --- | --- | --- | --- |
| Fatty acid binding protein 4 | FABP4 | P15090 | PWV | 18.45 | 1.1x10^-5^ | 18.37 | 0.00039 | 14.92 | 0.0010 | No |
| Tumor necrosis factor receptor 1 | TNFR1 | P19438 | PWV | 44.89 | 3.5x10^-6^ | 41.35 | 0.00039 | 45.02 | 0.0037 | No |
| Urokinase receptor | UPAR | Q03405 | PWV | 14.55 | 4.5x10^-5^ | 14.98 | 0.00092 | 16.43 | 0.0051 | No |
| Lymphotoxin beta receptor | LTBR | P36941 | PWV | 3.32 | 1.1x10^-5^ | 2.96 | 0.0012 | 3.37 | 0.0051 | Yes |
| Tumor necrosis factor superfamily 14 | TNFRSF14 | Q92956 | PWV | 10.77 | 0.00067 | 12.47 | 0.0012 | 13.42 | 0.0083 | Yes |
| Ephrin type-B receptor 4 | EPHB4 | P54760 | PWV | 8.57 | 3.6x10^-5^ | 7.44 | 0.0023 | 8.88 | 0.0083 | Yes |
| Galectin 4 | GAL4 | P56470 | PWV | 5.78 | 1.2x10^-5^ | 5.18 | 0.0012 | 5.29 | 0.0086 | Yes |
| Tumor necrosis factor receptor 2 | TNFR2 | P20333 | PWV | 17.37 | 2.4x10^-5^ | 14.76 | 0.0023 | 16.30 | 0.010 | No |
| Trefoil factor family 3 | TFF3 | Q07654 | PWV | 13.93 | 1.1x10^-5^ | 11.10 | 0.0020 | 11.85 | 0.011 | Yes |
| Peptidoglycan recognition protein 1 | PGLYRP1 | O75594 | PWV | 55.83 | 0.0025 | 64.35 | 0.0033 | 70.64 | 0.015 | Yes |
| Delta homolog 1 | DLK1 | P80370 | PWV | 23.09 | 0.00013 | 21.20 | 0.0027 | 21.30 | 0.028 | Yes |
| Cystatin B | CSTB | P04080 | PWV | 5.15 | 0.00043 | 5.36 | 0.0020 | 4.88 | 0.039 | Yes |
| Kallikrein-6 | KLK6 | Q92876 | PWV | 1.17 | 0.0012 | 1.06 | 0.015 | 1.20 | 0.044 | Yes |
| Tumor necrosis factor receptor superfamily 10c | TNFRSF10C | O14798 | PWV | 12.40 | 0.011 | 11.30 | 0.053 | 15.76 | 0.044 | Yes |
| Src homology 2 domain-containing protein tyrosine phosphatase substrate 1 | SHPS1 | P78324 | PWV | 2.50 | 0.0015 | 2.57 | 0.0062 | 2.55 | 0.045 | Yes |
| Matrix metalloproteinase-9 | MMP9 | P14780 | PWV | 10.04 | 0.029 | 13.44 | 0.013 | 13.99 | 0.046 | No |
| N-terminal prohormone of brain natriuretic peptide | NTproBNP | P16860 | PWV | 9.43 | 1.8x10^-5^ | 8.05 | 0.0020 | 6.92 | 0.046 | No |
| Peptidase inhibitor 3 | PI3 | P19957 | PWV | 3.05 | 3.0x10^-5^ | 2.34 | 0.0062 | 2.31 | 0.046 | Yes |
| Cluster of differentiation 163 | CD163 | Q86VB7 | PWV | 36.74 | 0.0012 | 39.21 | 0.0033 | 34.60 | 0.054 | -- |
| Galectin 3 | GAL3 | P17931 | PWV | 1.73 | 0.00075 | 1.38 | 0.025 | 15.4 | 0.059 | -- |
| Insulin-like growth factor-binding protein 7 | IGFBP7 | P08833 | PWV | 48.95 | 0.0021 | 43.18 | 0.025 | 46.80 | 0.062 | -- |
| Insulin-like growth factor-binding protein 2 | IGFBP2 | P00750 | PWV | 47.67 | 0.0016 | 5.76 | 0.69 | 37.80 | 0.062 | -- |
| Cathepsin Z | CTSZ | Q9UBR2 | PWV | 5.35 | 0.00067 | 4.68 | 0.013 | 4.37 | 0.069 | -- |
| Cathepsin D | CTSD | P07339 | PWV | 0.92 | 0.0012 | 1.07 | 0.0020 | 0.82 | 0.069 | -- |
| Interleukin-18-binding protein | IL18BP | O95998 | PWV | 9.07 | 0.00090 | 7.20 | 0.026 | 7.67 | 0.069 | -- |
| Osteopontin | OPN | P10451 | PWV | 31.09 | 0.00057 | 25.08 | 0.019 | 24.17 | 0.076 | -- |
| Interleukin-2 receptor alpha | IL2RA | P01589 | PWV | 1.47 | 0.021 | 1.22 | 0.098 | 1.67 | 0.079 | -- |
| Interleukin 1 receptor, type I | IL1RT1 | P14778 | PWV | 10.33 | 0.0033 | 9.50 | 0.025 | 8.52 | 0.12 | -- |
| Tissue-type plasminogen activator | TPA | P00750 | PWV | 20.63 | 0.0043 | 27.17 | 0.0020 | 14.81 | 0.15 | -- |
| Integrin beta 2 | ITGB2 | P05107 | PWV | 3.94 | 0.030 | 5.76 | 0.0062 | 3.88 | 0.15 | -- |
| Chemokine ligand 16 | CXCL16 | Q9H2A7 | PWV | 4.27 | 0.049 | 4.43 | 0.080 | 4.32 | 0.18 | -- |
| Spondin1 | SPON1 | Q9HCB6 | PWV | 0.80 | 0.00055 | 0.53 | 0.052 | 0.41 | 0.24 | -- |
| Chitinase-3-like protein 1 | CHI3L1 | P36222 | PWV | 12.25 | 0.00029 | 8.40 | 0.034 | 5.70 | 0.25 | -- |
| Myoglobin | MB | P02144 | PWV | 70.23 | 0.0048 | 49.28 | 0.094 | 44.92 | 0.26 | -- |
| Tartrate resistant acid phosphatase | TRAP | P13686 | PWV | 1.58 | 0.029 | 1.99 | 0.019 | 1.15 | 0.29 | -- |
| Metalloproteinase inhibitor 4 | TIMP4 | Q99727 | PWV | 2.87 | 0.0028 | 1.52 | 0.17 | 1.48 | 0.29 | -- |
| Phospholipase C | PLC | P98160 | PWV | 19.39 | 0.0089 | 12.12 | 0.16 | 9.20 | 0.39 | -- |
| von Willebrand factor | VWF | Q9HCB6 | PWV | 29.87 | 0.0070 | 12.96 | 0.31 | 13.90 | 0.39 | -- |
| C-C motif chemokine ligand 15 | CCL15 | Q16663 | PWV | 31.06 | 0.033 | 23.17 | 0.17 | 12.34 | 0.57 | -- |
| Matrix metalloproteinase-2 | MMP2 | P08253 | PWV | 1.84 | 0.021 | 1.10 | 0.22 | 0.68 | 0.57 | -- |
| Neurogenic locus notch homolog protein 3 | NOTCH3 | Q9UM47 | PWV | 10.36 | 0.016 | 4.37 | 0.36 | 2.90 | 0.66 | -- |
| Osteoprotegerin | OPG | O00300 | PWV | 4.48 | 0.0043 | 0.95 | 0.57 | -0.63 | 0.80 | -- |
| Insulin-like growth factor-binding protein 1 | IGFBP1 | P08833 | PWV | 10.56 | 0.029 | -1.60 | 0.76 | 0.66 | 0.90 | -- |

p-values are adjusted via the Benjamini-Hochberg correction to control false discovery rate (FDR = 0.05)

Log2(fold change) is scaled to 1 standard deviation increase in outcome (PWV SD = 348.93)

**3b. Coronary artery calcification (continuous)**

| Protein name | Protein abbreviation | Protein symbol | Outcome | Model 1 log_2_ fold change | Model 1 p | Model 2 log_2_ fold change | Model 2 p | Full Model log_2_ fold change | Full Model p | Novel? |
| --- | --- | --- | --- | --- | --- | --- | --- | --- | --- | --- |
| Cystatin B | CSTB | P04080 | CAC (cont) | 5.61 | 0.00035 | 5.72 | 0.0038 | 5.30 | 0.019 | Yes |
| Growth differentiation factor 15 | GDF15 | Q99988 | CAC (cont) | 37.34 | 0.00027 | 26.15 | 0.055 | 27.66 | 0.11 | -- |
| Carboxypeptidase A1 | CPA1 | P15085 | CAC (cont) | 17.53 | 0.019 | 20.15 | 0.049 | 17.94 | 0.22 | -- |
| Osteoprotegerin | OPG | P15085 | CAC (cont) | 6.34 | 0.00027 | 3.70 | 0.11 | 3.87 | 0.23 | -- |
| Galectin 4 | GAL4 | P15085 | CAC (cont) | 4.73 | 0.0011 | 3.88 | 0.049 | 3.12 | 0.23 | -- |
| Fatty acid binding protein 4 | FABP4 | P15090 | CAC (cont) | 14.23 | 0.0021 | 12.81 | 0.049 | 8.70 | 0.23 | -- |
| Fas cell surface death receptor | FAS | P15090 | CAC (cont) | 23.00 | 0.013 | 21.84 | 0.10 | 19.81 | 0.23 | -- |
| N-terminal prohormone of brain natriuretic peptide | NTproBNP | P16860 | CAC (cont) | 7.13 | 0.0030 | 5.30 | 0.11 | 5.02 | 0.23 | -- |
| Tumor necrosis factor receptor 2 | TNFR2 | P20333 | CAC (cont) | 13.45 | 0.0030 | 10.07 | 0.11 | 9.03 | 0.27 | -- |
| Tumor necrosis factor receptor 1 | TNFR1 | P19438 | CAC (cont) | 29.85 | 0.0030 | 22.79 | 0.11 | 19.73 | 0.28 | -- |
| Trefoil factor family 3 | TFF3 | P19438 | CAC (cont) | 9.91 | 0.0030 | 6.44 | 0.22 | 5.56 | 0.46 | -- |
| Lymphotoxin beta receptor | LTBR | P36941 | CAC (cont) | 2.13 | 0.011 | 1.50 | 0.23 | 1.41 | 0.46 | -- |
| Cathepsin Z | CTSZ | Q9UBR2 | CAC (cont) | 4.44 | 0.011 | 3.56 | 0.16 | 2.93 | 0.46 | -- |
| Delta homolog 1 | DLK1 | Q9UBR2 | CAC (cont) | 15.69 | 0.022 | 12.05 | 0.23 | 10.88 | 0.46 | -- |
| Ephrin type-B receptor 4 | EPHB4 | P54760 | CAC (cont) | 5.20 | 0.027 | 3.38 | 0.35 | 3.15 | 0.60 | -- |
| Peptidase inhibitor 3 | PI3 | P19957 | CAC (cont) | 2.17 | 0.0085 | 1.33 | 0.28 | 1.07 | 0.61 | -- |
| Galectin 3 | GAL3 | P17931 | CAC (cont) | 1.29 | 0.027 | 0.88 | 0.33 | 0.74 | 0.62 | -- |
| Interleukin-18-binding protein | IL18BP | O95998 | CAC (cont) | 6.16 | 0.048 | 3.82 | 0.43 | 3.24 | 0.68 | -- |
| von Willebrand Factor | VWF | P04275 | CAC (cont) | 27.53 | 0.027 | 12.51 | 0.51 | 8.57 | 0.83 | -- |
| Spondin 1 | SPON1 | Q9HCB6 | CAC (cont) | 0.57 | 0.027 | 0.28 | 0.48 | 0.22 | 0.83 | -- |
| Chitinase-3-like protein 1 | CHI3L1 | P36222 | CAC (cont) | 7.61 | 0.048 | 3.23 | 0.61 | 1.42 | 0.90 | -- |
| Insulin-like growth factor-binding protein 1 | IGFBP1 | P08833 | CAC (cont) | 11.43 | 0.031 | 1.45 | 0.88 | 0.53 | 0.97 | -- |

p-values are adjusted via the Benjamini-Hochberg correction to control false discovery rate (FDR = 0.05)

Log2(fold change) is scaled to 1 standard deviation increase in outcome (CAC SD = 2.36)

**3c. Abdominal aortic calcification (continuous)**

| Protein name | Protein abbreviation | Protein symbol | Outcome | Model 1 log_2_ fold change | Model 1 p | Model 2 log_2_ fold change | Model 2 p | Full Model log_2_ fold change | Full Model p | Novel? |
| --- | --- | --- | --- | --- | --- | --- | --- | --- | --- | --- |
| Cystatin B | CSTB | P04080 | AAC (cont) | 4.99 | 0.0077 | 4.90 | 0.034 | 4.32 | 0.24 | -- |
| Tumor necrosis factor receptor 2 | TNFR2 | P36222 | AAC (cont) | 12.93 | 0.0077 | 9.69 | 0.17 | 10.76 | 0.28 | -- |
| Myoglobin | MB | P02144 | AAC (cont) | 81.91 | 0.0077 | 65.95 | 0.11 | 53.44 | 0.28 | -- |
| Growth differentiation factor 15 | GDF15 | Q99988 | AAC (cont) | 27.49 | 0.011 | 15.62 | 0.24 | 18.18 | 0.28 | -- |
| Peptidase inhibitor 3 | PI3 | P19957 | AAC (cont) | 2.15 | 0.011 | 1.38 | 0.22 | 1.47 | 0.28 | -- |
| Tumor necrosis factor receptor 1 | TNFR1 | P19438 | AAC (cont) | 27.19 | 0.011 | 20.14 | 0.22 | 21.41 | 0.28 | -- |
| Trefoil factor family 3 | TFF3 | Q07654 | AAC (cont) | 8.87 | 0.012 | 5.53 | 0.22 | 6.37 | 0.28 | -- |
| Urokinase receptor | UPAR | Q03405 | AAC (cont) | 10.10 | 0.014 | 9.12 | 0.12 | 7.79 | 0.28 | -- |
| Galectin 4 | GAL4 | P56470 | AAC (cont) | 3.68 | 0.014 | 2.70 | 0.22 | 2.82 | 0.28 | -- |
| Ephrin type-B receptor 4 | EPHB4 | P54760 | AAC (cont) | 5.55 | 0.020 | 3.93 | 0.22 | 4.61 | 0.28 | -- |
| Interleukin-18-binding protein | IL18BP | O95998 | AAC (cont) | 6.97 | 0.024 | 4.95 | 0.22 | 5.48 | 0.28 | -- |
| N-terminal prohormone of brain natriuretic peptide | NTproBNP | P16860 | AAC (cont) | 5.52 | 0.026 | 3.55 | 0.24 | 4.55 | 0.28 | -- |
| Interleukin 1 receptor, type I | IL1RT1 | P14778 | AAC (cont) | 8.69 | 0.028 | 7.42 | 0.22 | 7.19 | 0.28 | -- |
| Lymphotoxin beta receptor | LTBR | P36941 | AAC (cont) | 1.82 | 0.029 | 1.19 | 0.24 | 1.39 | 0.32 | -- |
| Thyroid hormone receptor | TR | P02786 | AAC (cont) | 3.54 | 0.024 | 3.64 | 0.11 | 2.36 | 0.34 | -- |
| Osteoprotegerin | OPG | O00300 | AAC (cont) | 5.00 | 0.0077 | 2.40 | 0.24 | 2.52 | 0.38 | -- |
| Galectin 3 | GAL3 | P17931 | AAC (cont) | 1.34 | 0.022 | 0.97 | 0.22 | 0.72 | 0.45 | -- |
| Spondin 1 | SPON1 | Q9HCB6 | AAC (cont) | 0.62 | 0.018 | 0.37 | 0.24 | 0.31 | 0.45 | -- |
| Cathepsin Z | CTSZ | Q9UBR2 | AAC (cont) | 3.75 | 0.032 | 2.81 | 0.22 | 1.98 | 0.48 | -- |
| Phospholipase C | PLC | P98160 | AAC (cont) | 18.14 | 0.028 | 11.99 | 0.24 | 8.13 | 0.55 | -- |
| Metalloproteinase inhibitor 4 | TIMP4 | Q99727 | AAC (cont) | 2.26 | 0.034 | 1.09 | 0.37 | 0.96 | 0.58 | -- |
| Fatty acid binding protein 4 | FABP4 | P15090 | AAC (cont) | 13.26 | 0.0077 | 11.64 | 0.11 | 3.26 | 0.63 | -- |
| Chitinase-3-like protein 1 | CHI3L1 | P36222 | AAC (cont) | 7.88 | 0.034 | 3.98 | 0.36 | 2.18 | 0.65 | -- |

p-values are adjusted via the Benjamini-Hochberg correction to control false discovery rate (FDR = 0.05)

Log2(fold change) is scaled to 1 standard deviation increase in outcome (AAC SD = 3.11)

**3d. Coronary artery calcification (binary)**

| Protein name | Protein abbreviation | Protein symbol | Outcome | Model 1 log_2_ fold change | Model 1 p | Model 2 log_2_ fold change | Model 2 p | Full Model log_2_ fold change | Full Model p | Novel? |
| --- | --- | --- | --- | --- | --- | --- | --- | --- | --- | --- |
| Cystatin B | CSTB | P04080 | CAC presence | 10.73 | 0.014 | 10.56 | 0.053 | 8.37 | 0.40 | -- |
| Tumor necrosis factor receptor 2 | TNFR2 | P20333 | CAC presence | 26.50 | 0.019 | 18.70 | 0.48 | 16.97 | 0.74 | -- |
| Osteoprotegerin | OPG | O00300 | CAC presence | 10.66 | 0.019 | 4.51 | 0.55 | 4.52 | 0.74 | -- |
| Fatty acid binding protein 4 | FABP4 | P15090 | CAC presence | 26.78 | 0.019 | 22.78 | 0.30 | 11.87 | 0.74 | -- |
| Growth differentiation factor 15 | GDF15 | Q99988 | CAC presence | 58.82 | 0.019 | 30.83 | 0.52 | 24.20 | 0.74 | -- |
| Peptidase inhibitor 3 | PI3 | P19957 | CAC presence | 4.75 | 0.019 | 2.97 | 0.48 | 2.23 | 0.74 | -- |
| Galectin 4 | GAL4 | P56470 | CAC presence | 8.26 | 0.019 | 6.03 | 0.48 | 4.41 | 0.74 | -- |
| N-terminal prohormone of brain natriuretic peptide | NTproBNP | P16860 | CAC presence | 13.03 | 0.025 | 8.62 | 0.48 | 6.18 | 0.74 | -- |
| Tumor necrosis factor receptor 1 | TNFR1 | P19438 | CAC presence | 54.38 | 0.025 | 37.19 | 0.48 | 31.08 | 0.74 | -- |
| Cathepsin Z | CTSZ | Q9UBR2 | CAC presence | 8.92 | 0.030 | 6.86 | 0.48 | 5.18 | 0.74 | -- |
| Lymphotoxin beta receptor | LTBR | P36941 | CAC presence | 4.05 | 0.042 | 2.61 | 0.52 | 2.60 | 0.74 | -- |
| von Willebrand factor | VWF | P04275 | CAC presence | 64.80 | 0.025 | 33.80 | 0.55 | 26.52 | 0.75 | -- |
| Trefoil factor family 3 | TFF3 | Q07654 | CAC presence | 18.01 | 0.025 | 9.95 | 0.52 | 7.30 | 0.75 | -- |

p-values are adjusted via the Benjamini-Hochberg correction to control false discovery rate (FDR = 0.05)
